# Phasic Sleep Events Shape Cognitive Function after Traumatic Brain Injury: Implications for the Study of Sleep in Neurodevelopmental Disorders

**DOI:** 10.3934/neuroscience.2016.2.232

**Published:** 2016-06-28

**Authors:** Carolyn E. Jones, Miranda M. Lim

**Affiliations:** 1 Sleep Disorders Clinic, Division of Hospital and Specialty Medicine, Veterans Affairs Portland Health Care System, Portland, OR, USA;; 2 Departments of Medicine, Neurology and Behavioral Neuroscience, and Oregon Institute of Occupational Health Sciences, Oregon Health & Sciences University, Portland, OR, USA

## Abstract

The biological functions of sleep have long eluded the medical and research community. In four consecutive issues of *AIMS Neuroscience*, original and review manuscripts were recently published regarding the mechanisms and function of sleep. These articles highlight the well-timed topic of quantitative sleep markers and cognitive functioning as one of extensive interest within the field of neuroscience. Our commentary on the original research performed by Cote, Milner, and Speth (2015) brings attention to the importance of examining individual differences in sleep and cognition in subjects with traumatic brain injury (TBI), and provides support for conducting similar sleep analyses in neurodevelopmental disorders.

## 1. The Elusive Function of Sleep

We read with interest the past 4 issues of *AIMS Neuroscience*, where the timely topic of the biological function of sleep was reviewed over multiple issues (see [1–3]). From these reviews, it is evident that despite extensive knowledge of the cellular mechanisms and the neuroanatomical underpinnings of sleep, the function of sleep remains elusive, with the majority of research conclusions derived from observations of the immediate effects of acute or chronic sleep deprivation.

In their review, Zielinski, McKenna, and McCarley (2016) provide a comprehensive review on the underlying physiology of sleep regulation along with the cellular and molecular pathways activated or inhibited by sleep and wake. Barone and Krieger (2015) discuss the potential ecological purposes of sleep and provide an in-depth review of how sleep impacts overall health. Assefa and colleagues (2015) provide a concise history of sleep research and summarize what we know about the biological functions of sleep by reviewing mostly neurocognitive outcomes associated with sleep deprivation. Although each of these articles provides an accurate summary or the available data regarding sleep structure and function in adults, their back-to-back publications in *AIMS Neuroscience* stresses the challenges the field faces in providing answers regarding the function of sleep. Notably, none of these articles reviewed the literature on the function of sleep in early development.

## 2. Properties of Phasic Sleep Events Predict Cognitive Ability after TBI—Commentary on Cote, Milner, and Speth (2015)

Within these recent issues, highly relevant original research by Cote, Milner, and Speth (2015) highlighted the importance of individual differences when investigating sleep function in patients with traumatic brain injury (TBI). In a research article entitled “Altered Sleep Mechanisms following Traumatic Brain Injury and Relation to Waking Function”, Cote and colleagues investigated neurophysiological features of sleep in patients with traumatic brain injury using multiple night assessments (subjects spent three nights in testing). Importantly, the authors took into account TBI severity (as determined by length of unconsciousness after injury) when conducting analyses in order to parse out the influence of injury from specific characteristics of their sleep structure on waking cognitive functioning.

Consistent with other work [4] (for review see: [5]), Cote et al. found that patients with TBI demonstrated disrupted sleep. Specifically, patients with TBI reported longer times to fall asleep and less time spent sleeping. The polysomnograms confirmed increased latency to sleep times but indicated no difference in total sleep time when compared with healthy controls [6]. However, patients with TBI had significantly decreased delta power at all sleep stages compared with controls, which likely accounted for the subjective feeling of poor overall sleep. The research presented by Cote and colleagues (2015) explicitly explored properties of phasic sleep events by analyzing both spontaneous and stimulus evoked k-complexes and spindles, thought to reflect inhibition of information processing during sleep. Amongst many other findings, they showed that TBI patients went longer between spontaneous k-complexes (lower density) than controls and had fewer evoked k-complexes in response to stimulus presentation [6].

Within subjects, there was between night variability in multiple sleep domains (e.g. sigma power, spindle duration) that was only present in the TBI patient group. Other researchers have also noted intra-subject variability in sleep patterns across nights in patient populations (e.g. the elderly [7], children with attention-deficit/hyperactivity disorder (ADHD) [8]). This underscores the value of repeated testing and provides some perspective in interpreting data collected from a single night of sleep. Additionally, both the presence and overall spread of this within subject variation in certain populations is an outcome that is worth investigating in more depth as an indicator of sleep stability.

When presented in context with the three aforementioned articles reviewing the function of sleep, we found the work by Cote et al. especially interesting given their analysis of individual differences in sleep patterns and the relationship to waking cognitive function. They found that patients with more severe traumatic brain injury generated fewer *evoked* k-complexes (in response to auditory stimuli) without influencing *spontaneous* k-complex production. Injury severity was also negatively correlated to the amount of spindles produced (spindle density) [6]. This is consistent with other reports that sleep features may be associated with injury severity and functional outcomes after TBI [4].

By factoring injury severity and relevant properties of the observed sleep landscape into a multiple regression analysis, Cote and colleagues were able to conclude that spindle activity in patients with TBI predicted better performance on working memory and general cognitive functioning tests above and beyond the influence of the brain injury alone. This work provides a refreshing perspective to the field by examining how specific sleep events contribute to cognitive functioning.

It would be interesting to see how the phasic sleep events studied by Cote et al., specifically density of spindles and k-complexes, influence waking cognition in other patient populations. Although the immediate cognitive effects of sleep deprivation were reviewed in detail and the now well-established role of sleep in memory consolidation was succinctly described in Assefa et al. (2015), the potential use of sleep markers to predict cognitive performance at later times (an approach utilized by Cote et al.) was mostly overlooked in the review articles (but see [3]) and is an area of research that, when fully established, could allow us to uncover potential biomarkers of disease. Determining how the EEG morphology of phasic sleep events is related to waking cognition in neuropsychiatric disorders is an exciting area of research that deserves more attention in future studies.

## 3. Studying Sleep Structure across Development to Inform Function

The structure of sleep across the lifespan is always changing and the functional role of sleep events may differ in infancy compared to adulthood. Given the focus on the mechanisms of sleep regulation and health outcomes associated with sleep presented in the current set of reviews, it is surprising that age-related sleep changes were not discussed in more detail. Changes in the macrostructure of sleep have been well described over the past several decades [9–13] and sleep disturbances are a common complaint among elderly patients. Reductions in slow wave sleep and total sleep time, combined with frequent night awakenings characterize the sleep structure of the elderly. As humans age, the inhibitory phasic sleep events discussed in detail by Cote and colleagues (k-complexes and spindles) start to decline [14,15] and intra-individual variation across nights begins to increase [7,15].

Many neurodevelopmental disorders, including the autism spectrum disorders and ADHD, can be characterized by cognitive deficits in the same tests of working memory (e.g. digit span task) and general cognitive ability (e.g. digit symbol coding task) utilized in the research of Cote and colleagues. However, the role of sleep early in development was only mentioned in passing in two of the review articles [2,3] and not mentioned at all in the third review [1] published in the past 4 issues of *AIMS Neuroscience*. Normal sleep development includes the appearance of spindles during the first few months of life [16,17] in a pattern that matures along with the development of the thalamo-cortical neural circuitry [18,19] and may be an indicator of normal neural development [17,20–23]. Human and non-human animal research that investigates how phasic sleep parameters predict neurocognitive development in young subjects may help to considerably advance our understanding of prominent neurodevelopmental disorders and provide further understanding of how sleep shapes the developing brain.
